# DNA sequence variants in the LOXL1 gene are associated with pseudoexfoliation glaucoma in a U.S. clinic-based population with broad ethnic diversity

**DOI:** 10.1186/1471-2350-9-5

**Published:** 2008-02-06

**Authors:** Bao Jian Fan, Louis Pasquale, Cynthia L Grosskreutz, Douglas Rhee, Teresa Chen, Margaret M DeAngelis, Ivana Kim, Elizabeth del Bono, Joan W Miller, Tiansen Li, Jonathan L Haines, Janey L Wiggs

**Affiliations:** 1 Department of Ophthalmology, Harvard Medical School, Massachusetts Eye and Ear Infirmary, Boston, Massachusetts, USA; 2 Center for Human Genetics Research, Vanderbilt University School of Medicine, Nashville, Tennessee, USA

## Abstract

**Background:**

Pseudoexfoliation syndrome is a major risk factor for glaucoma in many populations throughout the world. Using a U.S. clinic-based case control sample with broad ethnic diversity, we show that three common SNPs in LOXL1 previously associated with pseudoexfoliation in Nordic populations are significantly associated with pseudoexfoliation syndrome and pseudoexfoliation glaucoma.

**Methods:**

Three LOXL1 SNPs were genotyped in a patient sample (206 pseudoexfoliation, 331 primary open angle glaucoma, and 88 controls) from the Glaucoma Consultation Service at the Massachusetts Eye and Ear Infirmary. The SNPs were evaluation for association with pseudeoexfoliation syndrome, pseudoexfoliation glaucoma, and primary open angle glaucoma.

**Results:**

The strongest association was found for the G allele of marker rs3825942 (G153D) with a frequency of 99% in pseudoexfoliation patients (with and without glaucoma) compared with 79% in controls (p = 1.6 × 10^-15^; OR = 20.93, 95%CI: 8.06, 54.39). The homozygous GG genotype is also associated with pseudoexfoliation when compared to controls (p = 1.2 × 10^-12^; OR = 23.57, 95%CI: 7.95, 69.85). None of the SNPs were significantly associated with primary open angle glaucoma.

**Conclusion:**

The pseudoexfoliation syndrome is a common cause of glaucoma. These results indicate that the G153D LOXL1 variant is significantly associated with an increased risk of pseudoexfoliation and pseudoexfoliation glaucoma in an ethnically diverse patient population from the Northeastern United States. Given the high prevalence of pseudooexfoliation in this geographic region, these results also indicate that the G153D LOXL1 variant is a significant risk factor for adult-onset glaucoma in this clinic based population.

## Background

Glaucoma is one of the leading causes of blindness worldwide and results in an irreversible degeneration of the optic nerve that is usually associated with a high intraocular pressure. The disease is clinically and genetically heterogeneous with early onset forms exhibiting mendelian inheritance while the common late onset forms have a more complex genetic architecture. Several genes have been associated with the mendelian forms of early-onset glaucoma: defects in the myocilin gene (GLC1A) primarily cause elevated pressure [1,2], the optineurin gene (GLC1E), appears to contribute to disease in familial low tension glaucoma [3-5]; and the WDR36 gene (GLC1G) may be a modifier gene that influences the severity of the glaucoma phenotype [6,7]. Seven other glaucoma gene loci (GLC1B, GLC1C, GLC1D, GLC1F, GLC1H, GLC1J, GLC1K) have been identified using large affected pedigrees and Mendelian linkage approaches [8-15].

Primary open angle glaucoma (POAG) and glaucoma associated with pseudoexfoliation (PXF, also called exfoliation) are the most common types of adult onset glaucoma. A positive family history is a major risk factor for these conditions, which suggests that specific gene defects are likely to contribute [16-20]; however, the genetic contributions to these disorders are complex and are likely to involve multiple genetic factors and possibly the influence of environmental exposures. Genome studies have been completed for POAG but as yet genes conferring significant susceptibility have not been identified [18,21-23].

Pseudoexfoliation syndrome results in the deposition of microfibrillar material throughout the eye. In over 50% of cases this condition is associated with glaucoma characterized by elevated intraocular pressure and optic nerve degeneration [24]. The nature of the deposited microfibrillar material is not known, and in addition to the eye it has also been identified in systemic tissues including renal vasculature, skin and liver [25]. Unlike other forms of glaucoma, systemic conditions have also been associated with pseudoexfoliation including abdominal aortic aneurysm [26], and elevated levels of serum homocysteine [27].

Familial aggregation studies have suggested a significant genetic contribution to pseudoexfoliation glaucoma [16,17,19], and one genome scan has recently been published [28]. However, the late age of onset makes genetic approaches using multigenerational families difficult. Recently, a whole genome association study using a collection of cases and controls from Iceland and a second population from Sweden identified a strong association between single nucleotide polymorphisms (SNPs) in the LOXL1 gene in patients with pseudoexfoliation [29]. Three SNPs were found to be associated with the disease: two nonsynonomous SNPs located within exon 1: rs1048661 (R141L) and rs3825942 (G153D) and rs2165241 located in the first intron of the gene. All three of these SNPs were in significant linkage disequilibrium (LD) in the studied population. This study reported odd ratios of 2.46 for allele G of rs1048661 and 20.10 for allele G of rs3825942 and 3.62 for allele T of rs2165241. Taken together these SNPs account for 99% of pseudoexfoliation cases in this homogenous population.

Independent replication studies are required to accurately assess the contribution of the associated alleles to complex disease, and in this case replication is especially important because the studied Nordic population has a limited gene pool and a very high prevalence of pseudoexfoliation [19,30]. To determine if the results from the Nordic study are generalizable to a heterogeneous population from the North eastern United States, we used an existing sample (206 PXF, 331 POAG, and 88 controls) from the Glaucoma Consultation Service at the Massachusetts Eye and Ear Infirmary to provide independent estimates of the association of LOXL1 SNPs with the pseudoexfoliation syndrome and pseudoexfoliation glaucoma.

## Methods

### Patient population

206 PXF patients, 331 POAG patients, and 88 control individuals were identified from the Glaucoma Consultation Service at the Massachusetts Eye and Ear Infirmary for this study. All patients and controls were recruited after informed consent, and the study was approved by the Massachusetts Eye and Ear Infirmary Institutional Review Board. For this patient population, glaucoma was defined as elevation of intraocular pressure and optic nerve disease as previously described [18]. Patients with PXF were identified by the presence of the characteristic fibrillar material present in the ocular anterior segment. Patients with iris transillumination defects without the presence of the fibrillar material were not were not identified as pseudoexfoliation patients, POAG patients or controls. Controls did not have fibrillar material, iris changes or evidence of glaucoma. Of the 206 patients with pseudoexfoliation, 146 also had glaucoma. The POAG patients had elevated intraocular pressure and optic nerve changes consistent with glaucoma, but did not have any evidence of psesudoexfoliation. The average age of the POAG patients and the PXF patients was 75. Because of the age-dependence of the pseudoexfoliation syndrome, only controls older than age 60 were used for this analysis with an average age of 72. The patients and controls were primarily Caucasian of European ancestry but the studied population also included African-Americans (6% of PXF, 22% of POAG and 6% of controls). 58% of the patients were female with 42% male, while 57% of the controls were female and 43% were male.

### Genotyping

Genotyping for rs2165241 and rs3825942 was performed for all samples using ABI TaqMan assays (assay by demand) performed according to the manufacturers instructions. rs1048661 was genotyped using direct sequencing, and the genotypes for all three SNPs were confirmed by direct genomic sequencing of a random group of samples (Table 1). For sequencing, products from PCR amplification were purified and sequenced using ABI BIG DYE chemistries and an automated ABI 3100 genetic analyzer. Sequence data was analyzed using the Vector NTI software package. The primer sequences and PCR conditions for LOXL1 sequencing are listed in Table 1. The observed genotypes of all 3 SNPs did not deviate from Hardy-Weinberg Equilibrium.

**Table 1:** Primer sequences and PCR conditions for LOXL1 sequencing

Exon	Primer Sequence	MgCl_2 _(mM)	Annealing Temperature (°C)	Amplicon (bp)
1A	F: TCCCAGCCTGTTGCTTATTC	1.5	55	326
	R: AGGCCTGGTGGACAGAGAG			
1B	F: AAGCAAGGAGCCTTCCTGTC	1.5	55	260
	R: GTACACCTGCCCGTTGTTCT			
1C*	F: GCAGGTGTACAGCTTGCTCA	1.5	55	464
	R: ACACGAAACCCTGGTCGTAG			
1D	F: CTCCTACCCGCAGCAGTTC	1.5	55	227
	R: GGTACTCGGGCAGCTCTTC			
1E	F: CGACCAGGGTTTCGTGTACT	1.5	55	280
	R: AGGGTAGGCCTGCTCGAAG			
1F	F: AGCAGGCCTACCCTGACC	1.5	55	340
	R: GCCTCCAGGAAGTTCTAAGGA			
2	F: GGAGGTCTCTGGGCATTAGC	1.5	58	354
	R: TCACTGATGAAACGGTCAGG			
3	F: TCTTCAGGGACAAGGAGTGG	1.5	58	357
	R: AGTGCTCATGGTGGGAGG			
4	F: CAGGGAAGACTAGGCCCTCT	1.5	58	324
	R: CTGTGAGCAGAGCTGAGTGG			
5	F: GAAACAAGCAGCATCACAGC	1.5	58	220
	R: GGCTGAAGCTTCTTTCAGGA			
6	F: TCTGGTGAGCAGTTGAGGTG	1.5	58	202
	R: GGTGGGCAAACTCTTACTGG			
7	F: CCCTCATTGACCCACTGTCT	1.5	58	356
	R: GCATGCAGAGCCACAGAGTA			

Note.-F: forward; R: reverse.

*The SNP rs1048661 is located in exon 1 and was analyzed in this amplicon.

### Statistical analysis

The observed genotype frequencies in the patients and controls were tested for Hardy-Weinberg equilibrium (HWE) and the difference between the observed and expected frequencies was tested for significance using chi-square test. Association analyses were performed using SAS statistical software (version 9.1.3; SAS Institute, Cary, NC). Fisher's exact test was used to compare allele or genotype frequencies of PXF or POAG patients with controls. The confidence intervals (CIs) for ORs were calculated by the logit method. Haplotype frequencies were estimated and tested using WHAP [31]. Conditional analysis based on haplotypes was performed using WHAP [31]. The LD analysis was performed using Haploview [32]. Multiple testing was adjusted using the Bonferroni correction.

## Results

### LOXL1 SNP association studies

Three common LOXL1 SNPs (rs1048661, rs3825942, and rs2165241) were used for these studies. Genotypes were determined for all three SNPs for the PXF patients, POAG patients and controls. The G allele of rs3825942 was found in a significantly higher frequency in pseudoexfoliation patients (with and without glaucoma) than in POAG patients or in control samples (99% in PXF, 77% in POAG and 79% in controls, p = 1.6 × 10^-15 ^(Table 2). The GG genotype was also significantly associated with pseudoexfoliation (98%; p = 1.2 × 10^-12^; OR = 23.57, 95%CI: 7.95, 69.85) compared with POAG patients (59%) and controls (68%). The T allele of the intronic SNP, rs2165241 was also significantly more frequent in pseudoexfoliation patients than in POAG patients or controls (p = 1.2 × 10^-11^) and the TT genotype was also significantly associated with the pseudoexfoliation condition (p = 5.0 × 10^-10^; OR = 4.14, 95%CI: 2.32, 7.39). In this population we did not find a highly significant association between pseudoexfoliation and the other nonsynonymous SNP (rs1048661, R141L) with significant association in the Nordic population.

**Table 2:** Distribution of LOXL1 sequence variants in PXF patients, POAG patients and Controls

		Genotype Frequency (%)				Allele Frequency (%)			
**rs1048661 (R141L)**	N	TT	TG	GG	*P*_value	T	G	*P*_value	OR G/T (95%CI)

**PXF**	199	6 (3.0)	56 (28.1)	137 (68.9)	0.011	68 (17.1)	330 (82.9)	0.005	1.90 (1.23, 2.93)
PXF (Glaucoma)	141	2 (1.4)	41 (29.1)	98 (69.5)	0.0028	45 (16.0)	237 (84.0)	0.0031	2.06 (1.29, 3.30)
PXF (No Glaucoma)	58	4 (6.9)	15 (25.9)	39 (67.2)	0.36	23 (19.8)	93 (80.2)	0.12	1.58 (0.89, 2.80)
**POAG**	304	29 (9.5)	110 (36.2)	165 (54.3)	0.84	168 (27.6)	440 (72.4)	0.92	1.02 (0.70, 1.51)
**Controls**	80	9 (11.3)	27 (33.7)	44 (55.0)		45 (28.1)	115 (71.9)		

**rs3825942 (G153D)**	N	AA	AG	GG	*P*_value	A	G	*P*_value	OR G/A (95%CI)

**PXF**	206	1 (0.5)	3 (1.5)	202 (98.0)	1.2 × 10^-12^	5 (1.2)	407 (98.8)	1.6 × 10^-15^	20.93 (8.06,54.39)
PXF (Glaucoma)	146	0 (0.0)	3 (2.1)	143 (97.9)	7.3 × 10^-11^	3 (1.0)	298 (99.0)	1.3 × 10^-13^	24.77 (7.50, 81.83)
PXF (No Glaucoma)	60	1 (1.7)	0 (0.0)	59 (98.3)	1.4 × 10^-6^	2 (1.7)	118 (98.3)	2.7 × 10^-7^	15.17 (3.58, 64.34)
**POAG**	325	17 (5.2)	115 (35.4)	193 (59.4)	0.044	149 (22.9)	501 (77.1)	0.54	0.86 (0.57, 1.30)
**Controls**	88	8 (9.1)	20 (22.7)	60 (68.2)		36 (20.5)	140 (79.5)		

**rs2165241 (intron)**	N	CC	CT	TT	*P*_value	C	T	*P*_value	OR T/C (95%CI)

**PXF**	200	12 (6.0)	72 (36.0)	116 (58.0)	5.0 × 10^-10^	96 (24.0)	304 (76.0)	1.2 × 10^-11^	3.77 (2.56, 5.55)
PXF (Glaucoma)	143	7 (4.9)	54 (37.8)	82 (57.3)	2.1 × 10^-9^	68 (23.8)	218 (76.2)	1.5 × 10^-10^	3.82 (2.53,5.78)
PXF (No Glaucoma)	57	5 (8.8)	18 (31.6)	34 (59.7)	4.0 × 10^-5^	28 (24.6)	86 (75.4)	1.0 × 10^-6^	3.66 (2.16, 6.21)
**POAG**	323	116 (35.9)	148 (45.8)	59 (18.3)	0.40	380 (58.8)	266 (41.2)	0.33	0.83 (0.59, 1.18)
**Controls**	80	27 (33.7)	33 (41.3)	20 (25.0)		87 (54.4)	73 (45.6)		

P values and OR are calculated when compared to controls. Association analyses were performed using SAS statistical software (version 9.1.3; SAS Institute, Cary, NC). Fisher's exact test was used to compare allele or genotype frequencies of PXF or POAG patients with controls. The confidence intervals (CIs) for ORs were calculated by the logit method. Hardy-Weinberg equilibrium was tested by chi-square test.

For this case control cohort, the attributable risk of the GG genotype for rs3825942 is approximately 99% (Table 2). Although our sample is primarily Caucasian patients of European ancestry the studied population also included African-Americans (6% of PXF, 22% of POAG and 6% of controls). The rs3825942 GG genotype was more frequent in African Americans with PXF in the sample (100% PXF, 40% POAG, 20% controls; p = 0.0007, Fisher's exact test), compared with African American POAG patients and controls although the sample size was too small to reach genome-wide statistical significance. The association with pseudoexfoliation was not different between patients with only the pseudoexofoliation syndrome compared with patients with pseudoexfoliation glaucoma, arguing that the LOXL1 protein participates in the syndrome and in the associated glaucoma. Interestingly, POAG is not associated with these LOXL1 SNPs (Table 2), which suggests that a different set of genetic factors contributes to this condition.

### Haplotype analyses and linkage disequilibrium

To determine if the rs3825942 association effect is independent of the other two SNPs, we investigated the haplotype associations for the patient sample. Haplotype frequencies were estimated and tested using WHAP [31]. The most common haplotype (TGG) consisting of all 3 risk alleles had significantly higher frequency in PXF patients than in controls (p = 2.8 × 10^-11^; OR = 3.66, 95%CI: 2.53, 5.30; Table 3). The association of rs3825942 with PXF was still significant after controlling for the effect of the other two SNPs (p = 0.002). This suggests that the effect of rs3825942 on PXF is independent of rs1048661 and rs2165241. Conversely, rs1048661 and rs2165241 were no longer associated with PXF after controlling for the other SNPs (p = 0.98 and 0.43 respectively). The two coding SNPs (rs1048661 and rs3825942) were reported to be in strong LD (D' = 1.0) in the Nordic population [29]. However, our analysis showed that they were in less LD in our heterogenous populations, especially in PXF patients (D' = 0.09, r^2 ^= 0).

**Table 3:** Haplotype analysis of rs1048661, rs3825942 and rs2165241 in PXF patients and Controls

	Estimated haplotype frequency (%)				
Haplotype	PXF	Controls	Combined	*P*_value	OR (95%CI)

GGT	75.3	45.4	66.3	2.8 × 10^-11^	3.66 (2.53, 5.30)
TGC	16.3	26.3	19.3	0.0057	0.54 (0.35, 0.83)
GAC	1.3	19.7	6.8	1.9 × 10^-12^	0.05 (0.02, 0.13)
GGC	6.6	7.2	6.8	0.78	0.88 (0.44, 1.74)
TGT	0.6	0.7	0.6	0.78	0.85 (0.08, 9.45)
GAT	0.0	0.6	0.2	0.11	-
TAC	0.0	0.0	0.0	1.00	-
TAT	0.0	0.0	0.0	1.00	-

Total	100.0	100.0	100.0	1.8 × 10^-11^*	-

*This *P *value was obtained from the omnibus test using WHAP,^31 ^while the other *P *values were obtained from the haplotype-specific test. OR was calculated for each individual haplotype compared to all the other haplotypes. 'GGT' is the combination of 'at risk' alleles at all three SNPs.

### LOXL1 sequence analyses

To determine if other common DNA sequence variants in LOXL1 contribute to the PXF phenotype, we sequenced all 7 exons of the LOXL1 gene and the flanking intronic regions in 7 randomly selected pseudoexfoliation patients. The genotypes for SNPs rs3825942, rs1048661, and rs2165241 were confirmed in this sample, however, we did not find other DNA sequence variants that were significantly associated with the condition.

## Discussion

The results of this study provide strong evidence for an association between DNA sequence variants in LOXL1 and pseudoexfoliation in a clinic-based population from the Northeastern United States. In this patient population the strongest association is found for a nonsysnonomous SNP, G152D, which is also one of the SNPs that demonstrated the strongest association in the Nordic population. Interestingly, the second nonsynonomous SNP showing significance in the Nordic population, R141L, is not significantly associated with pseduoexfoliation in our ethnically diverse clinic-based sample. This result probably reflects a difference in patterns of linkage disequilibrium between our ethnically heterogeneous patient population and the relatively homogeneous Nordic population. Very recently, associations between these LOXL1 SNPs and pseudoexfoliation patients in Australia [33] and the Midwestern US [34] have been reported. The results from Australia support an association that is not as strong as that seen in the Midwestern US or our study where the population attributable risk is near 90%. The Australian population has a nine-fold lower lifetime incidence of pseudoexfoliation compared to the Nordic populations and yet has similar distributions of LOXL1 genotypes between cases and controls. These results suggest that additional factors may influence the development of the pseudoexfoliation phenotype.

Loxl1 is one member of a family of proteins that catalyze the polymerization of tropoelastin to form the mature elastin polymer [35]. The protein also participates in elastin homeostasis and renewal, and is involved in spatially organizing elastogenesis at sites of elastin deposition. This function requires binding of LOXL1 to the elastin scaffold that is formed from fibrillin-containg microfibrils [36]. In a LOXL1 mouse knock-out abnormalities were detected in the skin and the aorta, two tissues reminiscent of reported findings in patients with psedueoxfoliation syndrome [35]. The ocular elastic tissues have not been completely characterized in the LOXL1 knock-out, and further investigations are ongoing. In pseudoexfoliation patients abnormal fibrillar material accumulates in the eye and in systemic tissues, and abnormal elastin formation has also been described in some tissues including the optic nerve [37,38]. Further characterization of tissues expected to be involved in glaucoma in the LOXL1 knock-out is currently underway.

The DNA sequence variants in LOXL1 that are associated with pseudoexfoliation are all located in the nonconserved N-terminal sequence that may have a regulatory function [35,36]. This portion of the protein may play a role in directing the LOXL1 protein to sites of elastogenesis, but is unlikely to affect the catalytic activity of the protein. Although the DNA sequence variant most significantly associated with the phenotype is a nonsynonymous SNP (G153D), the biological effect of this missense change has not been determined. It is possible that the variants associated with pseudoexfoliation are in linkage disequilibrium with other DNA sequence changes that affect gene expression, and further studies to investigate the level of LOXL1 mRNA and protein in ocular tissues from patients with pseudoexfoliation would be necessary to determine if a reduction in LOXL1 mRNA or protein is responsible for the condition. The genomic location of the LOXL1 gene overlaps in a head to tail fashion with a novel gene coding for an untranslated mRNA (FLJ31814), and all of the sequence variants known to be associated with pseudoexfoliation are located in the overlapped segment. This untranslated mRNA has reported ocular expression, although its function is completely unknown [39].

## Conclusion

This study demonstrates that DNA sequence variants in LOXL1 are strongly associated with pseudoexfoliation and pseudoexfoliation glaucoma in a heterogeneous clinic-based population with broad ethnic diversity. The strength of the association in this heterogeneous population is similar to that observed for pseudoexfoliation patients from the relatively homogenous Nordic population confirming the generalizability of this result. The variants associated with the disease are common and thus are also prevalent in the unaffected control sample. Given the complex inheritance of this condition it is highly likely that additional genetic factors and/or environmental exposures could influence the development of the disease. Other members of the LOX family as well as other proteins that contribute to the maintenance of elastic fibers and composition of the extracellular matrix are excellent candidates for additional genetic factors that could contribute to this common blinding disease.

## Competing interests

The author(s) declare that they have no competing interests.

## Authors' contributions

BJF carried out the molecular genetic studies and analyses and helped draft the manuscript, JLH participated in the association studies and haplotype analyses, TL contributed information regarding protein function and analyses in the LOXL1 knock-out mouse, LP, CLG, DR, TC, MD, IK, JWM all contributed patients for the study. ED coordinated patient examination and sample collection. JLW conceived of the study, and participated in its design and coordination and helped to draft the manuscript. All authors have read and approved the final manuscript.

## Pre-publication history

The pre-publication history for this paper can be accessed here:



## References

[B1] Stone EM, Fingert JH, Alward WL, Nguyen TD, Polansky JR, Sunden SL, Nishimura D, Clark AF, Nystuen A, Nichols BE, Mackey DA, Ritch R, Kalenak JW, Craven ER, Sheffield VC (1997). Identification of a gene that causes primary open angle glaucoma. Science.

[B2] Fingert JH, Stone EM, Sheffield VC, Alward WL (2002). Myocilin glaucoma. Ophthalmol.

[B3] Rezaie T, Child A, Hitchings R, Brice G, Miller L, Coca-Prados M, Heon E, Krupin T, Ritch R, Kreutzer D, Crick RP, Sarfarazi M (2002). Adult-onset primary open-angle glaucoma caused by mutations in optineurin. Science.

[B4] Aung T, Rezaie T, Okada K, Viswanathan AC, Child AH, Brice G, Bhattacharya SS, Lehmann OJ, Sarfarazi M, Hitchings RA (2005). Clinical features and course of patients with glaucoma with the E50K mutation in the optineurin gene. Invest Ophthalmol Vis Sci.

[B5] Hauser MA, Figueiredo Sena D, Flor JD, J Walter J, Auguste J, LaRocque KR, Graham FL, Del Bono E, Haines JL, Pericak-Vance MA, Allingham RR, Wiggs JL (2006). Distribution of optineurin sequence variations in an ethnically diverse population of low tension glaucoma patients from the United States. J of Glaucoma.

[B6] Hauser MA, Allingham RR, Linkroum K, Wang J, LaRocque-Abramson K, Figueiredo D, Santiago-Turla C, Del Bono EA, Haines JL, Pericak-Vance MA, Wiggs JL (2006). Distribution of WDR36 DNA sequence variants in primary open angle glaucoma patients. Invest Ophthal Vis Sci.

[B7] Monemi S, Spaeth G, DaSilva A, Popinchalk S, Ilitchev E, Liebmann J, Ritch R, Heon E, Crick RP, Child A, Sarfarazi M (2005). Identification of a novel adult-onset primary open-angle glaucoma (POAG) gene on 5q22.1. Hum Mol Genet.

[B8] Stoilova D, Child A, Trifan OC, Crick RP, Coakes RL, Sarfarazi M (1996). Localization of a locus (GLC1B) for adult-onset primary open angle glaucoma to the 2cen-q13 region. Genomics.

[B9] Wirtz MK, Samples JR, Kramer PL, Rust K, Topinka JR, Young J, Koler RD, Acott TS (1997). Mapping a gene for adult-onset primary open-angle glaucoma to chromosome 3q. Am J Hum Genet.

[B10] Trifan OC, Traboulsi EI, Stoilova D, Alozie I, Nguyen I, Rasa S, Sarfarazi M (1998). The third locus (GLC1D) for adult-onset primary open-angle glaucoma maps to the 8q23 region. Genomics.

[B11] Sarfarazi M, Child A, Stoilova D, Brice G, Desai T, Trifan OC, Poinoosawmy D, Crick RP (1998). Localization of the fourth locus (GLC1E) for adult-onset primary open angle glaucoma to the 10p15-p14 region. Am J Hum Genet.

[B12] Wirtz MK, Samples JR, Rust K, Lie J, Nordling L, Schilling K, Acott TS, Kramer PL (1999). GLC1F, a new primary open-angle glaucoma locus, maps to 7q35-q36. Arch Ophthalmol.

[B13] Wiggs JL, Maselli M, Lynch S, Yanagi G, DelBono E, Haines JL (2004). A genome-wide scan identifies novel early onset primary open angle glaucoma loci on 9q22 and 20p12. Am J Hum Genet.

[B14] Wang DY, Fan BJ, Chua JK, Tam PO, Leung CK, Lam DS, Pang CP (2006). A genome-wide scan maps a novel juvenile-onset primary open-angle glaucoma locus to 15q. Invest Ophthalmol Vis Sci.

[B15] Suriyapperuma SP, Child A, Desai T, Brice G, Kerr A, Crick RP, Sarfarazi M (2007). A new locus (GLC1H) for adult-onset primary open-angle glaucoma maps to the 2p15-p16 region. Arch Ophthalmol.

[B16] Budde WM, Jonas JB (1999). Family history of glaucoma in the primary and secondary open-angle glaucomas. Graefes Arch Clin Exp Ophthalmol.

[B17] Damji KF, Bains HS, Amjadi K, Dohadwala AA, Valberg JD, Chevrier R, Gould LF, Zackon DH, Addison DJ (1999). Familial occurrence of pseudoexfoliation in Canada. Can J Ophthalmol.

[B18] Wiggs JL, Allingham RR, Hossain A, Kern J, Auguste J, DelBono EA, Broomer B, Graham FL, Hauser M, Pericak-Vance M, Haines JL (2000). Genome-wide scan for adult onset primary open angle glaucoma. Hum Mol Genet.

[B19] Allingham RR, Loftsdottir M, Gottfredsdottir MS, Thorgeirsson E, Jonasson F, Sverisson T, Hodge WG, Damji KF, Stefansson E (2001). Pseudoexfoliation syndrome in Icelandic families. Br J Ophthalmol.

[B20] van Koolwijk LM, Despriet DD, van Duijn CM, Pardo Cortes LM, Vingerling JR, Aulchenko YS, Oostra BA, Klaver CC, Lemij HG (2007). Genetic contributions to glaucoma: heritability of intraocular pressure, retinal nerve fiber layer thickness, and optic disc morphology. Invest Ophthalmol Vis Sci.

[B21] Nemesure B, Jiao X, He Q, Leske MC, Wu SY, Hennis A, Mendell N, Redman J, Garchon HJ, Agarwala R, Schaffer AA, Hejtmancik F, Barbados Family Study Group (2003). A genome-wide scan for primary open-angle glaucoma (POAG): the Barbados Family Study of Open-Angle Glaucoma. Hum Genet.

[B22] Rotimi CN, Chen G, Adeyemo AA, Jones LS, Agyenim-Boateng K, Eghan BA, Zhou J, Doumatey A, Lashley K, Huang H, Fasanmade O, Akinsola FB, Ezepue F, Amoah A, Akafo S, Chen Y, Oli J, Johnson T (2006). Genomewide scan and fine mapping of quantitative trait loci for intraocular pressure on 5q and 14q in West Africans. Invest Ophthalmol Vis Sci.

[B23] Duggal P, Klein AP, Lee KE, Klein R, Klein BE, Bailey-Wilson JE (2007). Identification of novel genetic loci for intraocular pressure: a genomewide scan of the Beaver Dam Eye Study. Arch Ophthalmol.

[B24] Jeng SM, Karger RA, Hodge DO, Burke JP, Johnson DH, Good MS (2007). The risk of glaucoma in pseudoexfoliation syndrome. J Glaucoma.

[B25] Streeten BW, Li ZY, Wallace RN, Eagle RC, Keshgegian AA (1992). Pseudoexfoliative fibrillopathy in visceral organs of a patient with pseudoexfoliation syndrome. Arch Ophthalmol.

[B26] Schumacher S, Schlotzer-Schrehardt U, Martus P, Lang W, Naumann GO (2001). Pseudoexfoliation syndrome and aneurysms of the abdominal aorta. Lancet.

[B27] Roedl JB, Bleich S, Reulbach U, Rejdak R, Kornhuber J, Kruse FE, Schlotzer-Schrehardt U, Junemann AG (2007). Homocysteine in tear fluid of patients with pseudoexfoliation glaucoma. J Glaucoma.

[B28] Lemmela S, Forsman E, Sistonen P, Eriksson A, Forsius H, Jarvela I (2007). Genome-wide scan of exfoliation syndrome. Invest Ophthalmol Vis Sci.

[B29] Thorleifsson G, Magnusson KP, Sulem P, Walters GB, Gudbjartsson DF, Stefansson H, Jonsson T, Jonasdottir A, Jonasdottir A, Stefansdottir G, Masson G, Hardarson GA, Petursson H, Arnarsson A, Motallebipour M, Wallerman O, Wadelius C, Gulcher JR, Thorsteinsdottir U, Kong A, Jonasson F, Stefansson K (2007). Common sequence variants in the LOXL1 gene confer susceptibility to exfoliation glaucoma. Science.

[B30] Jonasson F, Damji KF, Arnarsson A, Sverrisson T, Wang L, Sasaki H, Sasaki K (2003). Prevalence of open-angle glaucoma in Iceland: Reykjavik Eye Study. Eye.

[B31] Purcell S, Daly MJ, Sham PC (2007). WHAP: haplotype-based association analysis. Bioinformatics.

[B32] Barrett JC, Fry B, Maller J, Daly MJ (2005). Haploview: analysis and visualization of LD and haplotype maps. Bioinformatics.

[B33] Hewitt AW, Sharma S, Burdon KP, Wang JJ, Baird PN, Dimasi DP, Mackey DA, Mitchell P, Craig JE Ancestral LOXL1 variants are associated with pseudoexfoliation in Caucasian Australians but with markedly lower penetrance than in Nordic people. Hum Mol Genet.

[B34] Fingert JH, Alward WL, Kwon YH, Wang K, Streb LM, Sheffield VC, Stone EM (2007). LOXL1 Mutations Are Associated with Exfoliation Syndrome in Patients from the Midwestern United States. Am J Ophthalmol.

[B35] Liu X, Zhao Y, Gao J, Pawlyk B, Starcher B, Spencer JA, Yanagisawa H, Zuo J, Li T (2004). Elastic fiber homeostasis requires lysyl oxidase-like 1 protein. Nat Genet.

[B36] Thomassin L, Werneck CC, Broekelmann TJ, Gleyzal C, Hornstra IK, Mecham RP, Sommer P (2005). The Pro-regions of lysyl oxidase and lysyl oxidase-like 1 are required for deposition onto elastic fibers. J Biol Chem.

[B37] Netland PA, Ye H, Streeten BW, Hernandez MR (1995). Elastosis of the lamina cribrosa in pseudoexfoliation syndrome with glaucoma. Ophthalmology.

[B38] Schlotzer-Schrehardt U, Dorfler S, Naumann GO (1992). Immunohistochemical localization of basement membrane components in pseudoexfoliation material of the lens capsule. Curr Eye Res.

[B39] Stanford S.O.U.R.C.E. http://source.stanford.edu.

